# Insights into the role of long non-coding RNAs in DNA methylation mediated transcriptional regulation

**DOI:** 10.3389/fmolb.2022.1067406

**Published:** 2022-12-02

**Authors:** Zhen Yang, Feng Xu, Andrew E. Teschendorff, Yi Zhao, Lei Yao, Jian Li, Yungang He

**Affiliations:** ^1^ Center for Medical Research and Innovation of Pudong Hospital, The Shanghai Key Laboratory of Medical Epigenetics, International Co-Laboratory of Medical Epigenetics and Metabolism, Ministry of Science and Technology, Institutes of Biomedical Sciences, Fudan University, Shanghai, China; ^2^ CAS Key Laboratory of Computational Biology, Shanghai Institute of Nutrition and Health, Chinese Academy of Sciences, University of Chinese Academy of Sciences, Shanghai, China; ^3^ Institute of Computing Technology, Chinese Academy of Sciences, Beijing, China; ^4^ Experiment Medicine Center, The Affiliated Hospital of Southwest Medical University, Luzhou, Sichuan, China; ^5^ Shanghai Fifth People’s Hospital, Fudan University, Shanghai, China

**Keywords:** lncRNA, circRNA, DNA methylation, histone modification, transcriptional regulation, regulatory network

## Abstract

DNA methylation is one of the most important epigenetic mechanisms that governing regulation of gene expression, aberrant DNA methylation patterns are strongly associated with human malignancies. Long non-coding RNAs (lncRNAs) have being discovered as a significant regulator on gene expression at the epigenetic level. Emerging evidences have indicated the intricate regulatory effects between lncRNAs and DNA methylation. On one hand, transcription of lncRNAs are controlled by the promoter methylation, which is similar to protein coding genes, on the other hand, lncRNA could interact with enzymes involved in DNA methylation to affect the methylation pattern of downstream genes, thus regulating their expression. In addition, circular RNAs (circRNAs) being an important class of noncoding RNA are also found to participate in this complex regulatory network. In this review, we summarize recent research progress on this crosstalk between lncRNA, circRNA, and DNA methylation as well as their potential functions in complex diseases including cancer. This work reveals a hidden layer for gene transcriptional regulation and enhances our understanding for epigenetics regarding detailed mechanisms on lncRNA regulatory function in human cancers.

## Introduction

DNA methylation is an epigenetic modification involving the transfer of the methyl group onto the C5 position of the cytosine at CpG dinucleotide sites to form the 5-methylcytosine (5mC). It has been widely recognized for DNA methylation as a major epigenetic mechanism in regulating gene expression, genome stability and cell fate (Deaton and Bird, 2011; Moore et al., 2013). DNA methylation at promoter region could determine the regulatory activity of the target genes by regulating chromatin accessibility and blocking recruitment of transcription factors (Blattler and Farnham, 2013; Hu et al., 2013). CpG islands within promoter regions are usually unmethylated and associated with a transcriptionally permissive state in normal physiology, whereas methylated CpG islands, which are often observed in cancer, generally associated with the closed chromatin configuration and lead to gene repression (Feinberg et al., 2006). DNA methylation status alterations are well known to influence transcript abundance of many cancer-related genes, thus may define different types of “driver” events, such as cell growth, proliferation, differentiation, and apoptosis processes (Borgel et al., 2010; Jones, 2012; Kulis et al., 2015; Fialkova et al., 2017).

DNA methylation is highly spatio-temporal specific across different cell types and developmental stages, and its emergence and maintenance are complex processes under precise regulation (Lister et al., 2009; Ziller et al., 2013). In mammalian cells, transfer of the methyl group to cytosine is catalyzed by three DNA methyltransferases (DNMTs): *DNMT3A*, *DNMT3B*, and *DNMT1*. It is recognized that *DNMT3A* and *DNMT3B* are *de novo* methyltransferases that establish DNA methylation patterns early in development, whereas *DNMT1* functions to preserve DNA methylation patterns from parental to daughter strand during every DNA replication cycle (Lyko, 2018). DNA demethylation is mainly mediated by the Ten-eleven translocation (TET) family members (*TET1*, *TET2*, and *TET3*). These enzymes are responsible for the hydroxylation of 5mC and its further oxidation, which finally get replaced by cytosine following base excision repair (Melamed et al., 2018). The DNA methylation status at particular site is not only determined by activity of DNMTs, which present limited sequence specificity (Furuta et al., 2014), but is also affected by coordinated function of other complexes, particularly chromatin-remodeling complexes and histone modification enzymes (Hervouet et al., 2018). For instance, it has been found that the maintenance of DNA methylation in heterochromatin requires the DNMT1/HDAC1 interaction and deacetylation state of histones, and the presence of 5mC is often correlated with histone deacetylation (Fuks et al., 2000). The Ubiquitin-like containing PHD Ring Finger 1 (UHRF1), which constitutes a complex with HDAC1, could interact with DNMT1 to promote DNA methylation inheritance during mid to late S phase (Liu et al., 2013; Nishiyama et al., 2020). Another example is the Polycomb Repressive Complex 2 (PRC2) protein EZH2, which has been shown to interact with DNMTs and is crucial for recruitment of DNMTs to specific loci (Vire et al., 2006; Wu et al., 2008). DNA hypermethylation observed in colon cancer could be partially regulated by interactions between DNMT3B and PRC1 or PRC2 (Jin et al., 2009). In recent years, accumulating evidence points towards long non-coding RNAs (lncRNAs) being an important piece in this jigsaw puzzle, representing a distinct class of epigenetic regulators that influence genome-wide DNA methylation patterns.

LncRNAs are defined as non-coding transcripts whose length ranges from 200 nt to more than 10 kb, and have been implicated in many physiological and pathological processes, including cancer (Cabili et al., 2011; Fatica and Bozzoni, 2014). A vast majority of lncRNAs are characterized as tissue and developmental stage specific with important functions in gene expression regulation, often act as competing endogenous RNA (ceRNA) to regulate the expression of downstream genes by binding to their common microRNA (miRNA) regulators (Ponting et al., 2009; Tay et al., 2014). In fact, lncRNAs could regulate gene expression *via* multiple mechanisms, including modulation of transcription, mRNA stability, translation and protein subcellular location by interacting with DNA, RNA or protein to form large complexes (Statello et al., 2021). Many lncRNAs act as scaffold or decoy to recruit or sequester other proteins or RNAs. They could affect chromatin architecture and genome organization to regulate gene expression by different mechanisms of action (Yao et al., 2019). Meanwhile, circular RNAs (circRNAs) being a new subtype of non-coding RNA formed by covalently closed loops through back splicing, now exhibit great potential with different cellular functions (Liu and Chen, 2022). They are involved in gene expression regulation by acting as sponge for miRNAs, or with other aspects of mechanisms. LncRNAs and circRNAs are widely implicated in the epigenetic regulatory mechanisms, such as DNA methylation and histone modification, and involved in the development and progression of many human malignancies (Hanly et al., 2018; Morselli and Dieci, 2022).

**FIGURE 1:**
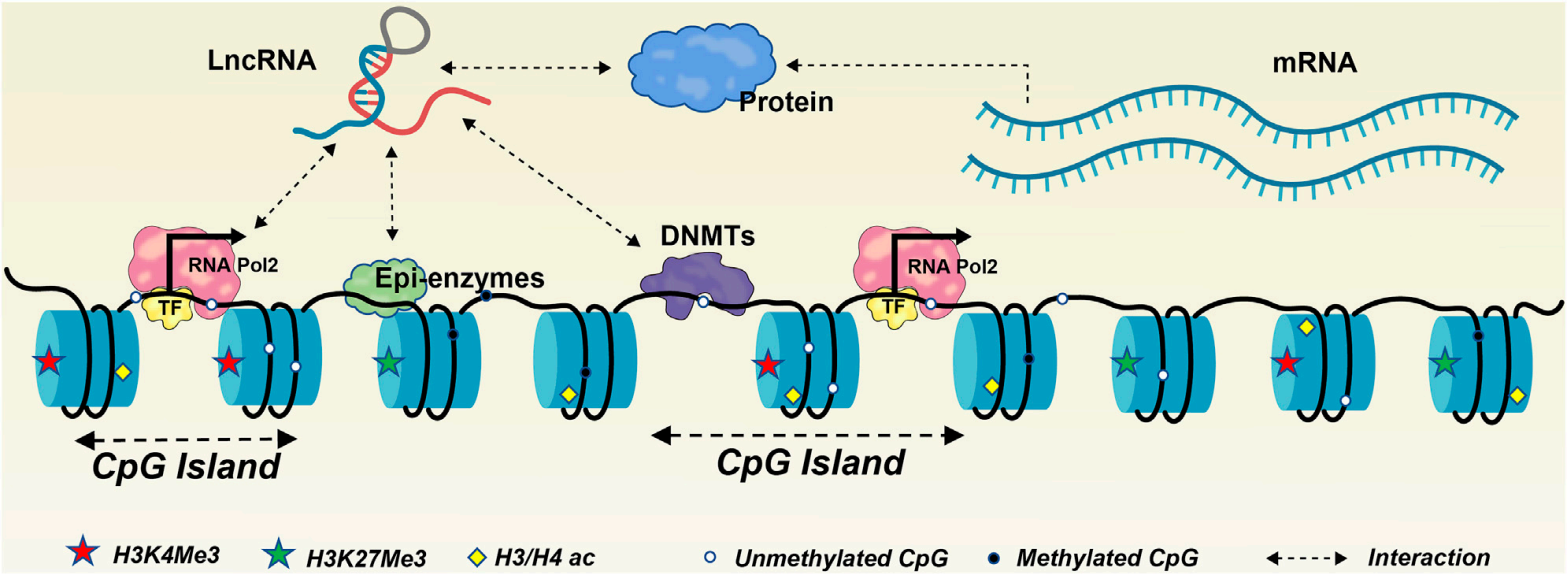
Complex regulatory network involving lncRNAs and DNA methylation. On one hand, DNA methylation change targeting promoters of lncRNA genes may affect its expression as observed for PCGs. On another hand, lncRNAs can modulate DNA methylation and transcription of proximal and distant genes by interacting with enzymes or proteins involved in epigenetic regulation.

Evidence has indicated that transcriptional control of lncRNAs and circRNAs are similar to that of protein-coding genes (PCGs), with their expression regulated by promoter methylation status (Wu et al., 2010; Li et al., 2015; Xu et al., 2018). On the other hand, studies also indicate that they are pivotal regulators modulating the epigenome by interacting with different epigenetic factors (Ferreira and Esteller, 2018). LncRNAs and circRNAs could regulate DNA methylation *via* interaction with DNMTs or other genes involved in chromatin organization, thereby regulating target gene expression in diverse biological processes (Mercer and Mattick, 2013). The dynamic nature of their repertoire and plasticity for lncRNAs and circRNAs in interacting with different molecules made this crosstalk between lncRNAs and DNA methylation a complex regulatory network to be elucidated at the system level (Figure 1). Therefore, a comprehensive review for achievements of the experimentally verified regulatory relationships among lncRNA, circRNA and DNA methylation is critically needed. Here we lay emphasis on those lncRNAs and circRNAs that have been identified to regulate DNA methylation with various mechanisms, as well as their roles in cancer development. Indeed, the broad phylogenies of lncRNAs and circRNAs and their important biological roles lead to the hypothesis that they could constitute another regulatory layer that shapes the epigenetic landscape, with great potential for diagnosis, prognosis, and personalized treatment of cancer.

## DNA methylation contributes to long non-coding RNA expression regulation

Beneath the aberrant cell proliferation of tumor formation is the complex interactions between a striking diversity of genetic and epigenetic factors, and the mechanisms of cancer development can be largely attributed to epimutations, which include the aberrant histone modifications and DNA hyper- and hypomethylation events across the genome (Banno et al., 2012). CpG hypermethylation is associated with specific chromatin conformation in blocking the recruitment of transcription factors, and generally promotes the transcription inhibition of tumor suppressor genes in cancer, whereas hypomethylation may lead to upregulation of oncogenes (Domcke et al., 2015). LncRNAs resemble mRNAs in length and biological characteristics but lack extended open reading frames (ORFs). Most of them are transcribed by RNA polymerase II, capped, polyadenylated, and often spliced, thus it is not surprise lncRNAs share similar epigenetic regulatory mechanisms with PCGs (Okazaki et al., 2002; Sati et al., 2012; Hangauer et al., 2013). This was confirmed by the observation of the lncRNA promoter methylation alterations in cancers (Yan et al., 2015), and also by the altered expression of numerous lncRNAs in response to the treatment with DNA methylation inhibitor 5-aza-2′-deoxycytidine (5-AZA-CdR) (Cao et al., 2016). Many lncRNAs that undergo cancer-associated methylation changes are found at the crossroads of key oncogenic pathways (Table 1). For example, a p53-induced lncRNA *TP53TG1* present promoter hypermethylation in gastric and colon cancers. This lncRNA was found to interact with the DNA/RNA binding protein YBX1, impede its nuclear localization and prevent YBX1-mediated activation of other oncogenes (Diaz-Lagares et al., 2016). Another example is the tumor suppressor lncRNA *GAS5* (Growth Arrest-Specific transcript 5), which was found downregulated in gastric cancer *via* promoter hypermethylation. This lncRNA plays a key role in adriamycin sensitivity, and represents a novel marker of prognosis and potential therapeutic target for gastric cancer (Sun et al., 2014; Zhang et al., 2016). LncRNA *CRNDE* presents promoter hypermethylation and downregulated expression in B lymphocytes of chronic lymphocytic leukemia (CLL) patients. It acts as a competing endogenous RNA (ceRNA) to repress miR-28, thereby regulating *NDRG2* expression. Overexpression of *CRNDE* by DNA methylation inhibitor 5-AZA-CdR promotes *NDRG2* expression, thereby inhibit cell proliferation and promote apoptosis in CLL (Ni et al., 2021).

**TABLE 1:** Representative lncRNAs whose expression regulated by promoter methylation.

LncRNA name	Methylation pattern	Tissue/disease	Target	Function	References
TP53TG1	Hypermethylation	Gastric cancer; colon cancer	YBX1	Cellular death resistance	Diaz-Lagares et al. (2016)
GAS5	Hypermethylation	Gastric cancer		Cell proliferation promotion	Sun et al. (2014); Zhang et al. (2016)
CRNDE	Hypermethylation	Chronic lymphocytic leukemia	miR-28	Competing endogenous RNA, cell proliferation promotion	Ni et al. (2021)
H19	Hypomethylation	Bladder cancer			Takai et al. (2001)
H19	Hypomethylation	Colorectal cancer			Tian et al. (2012)
H19	Hypomethylation	Oral squamous cell carcinoma			Lee et al. (2021)
H19	Hypermethylation	Peripheral blood of gastric cancer patients			Hu et al. (2021)
PlncRNA-1	Hypomethylation	Breast cancer	miR-136	Competing endogenous RNA, epithelial–mesenchymal transition (EMT)	Kang et al. (2020)
Esrp2-as	Hypomethylation	Breast cancer		Cell motility and proliferation promotion	Heilmann et al. (2017)
HNF1A-AS1	Hypermethylation	Laryngeal squamous cell carcinoma			Shi et al. (2020)
LINC00299	Hypermethylation	Breast cancer (TNBC)			Manoochehri et al. (2020)
LINC00472	Hypermethylation	Gastric cancer			Tsai et al. (2019)
RP11-713P17.4	Hypermethylation	Breast cancer			Pangeni et al. (2022)
SNHG12	Hypermethylation	Glioblastoma	miR-129-5p	Competing endogenous RNA	Lu et al. (2020)
SNHG11	Hypermethylation	Colorectal cancer		Promote CRC cell migration and metastasis under hypoxia	Xu et al. (2020)
CCND2 AS1	Hypomethylation	Cervical cancer		Inhibited the proliferation and cell cycle progression	Zhao et al. (2020a)
SOX21-AS1	Hypomethylation	Cervical cancer		Regulation of the Wnt signaling pathway	Du et al. (2021a)
H19	Hypomethylation	Nasopharyngeal carcinoma			Ng et al. (2003)
H19	Hypomethylation	Colorectal cancer			Cui et al. (2002)
H19	Hypermethylation	Cervical cancer			Roychowdhury et al. (2020)
MEG3	Hypermethylation	Esophageal squamous cell carcinoma	miR-9	Competing endogenous RNA, promote cell proliferation and invasion	Dong et al. (2017)
PLUT	Hypermethylation	Lung adenocarcinoma			Kim-Wanner et al. (2020)
LINC00473	Hypermethylation	Colorectal cancer			Ruiz-Banobre et al. (2022)
MEG3	Hypermethylation	Breast cancer			Pan et al. (2022)
LINC00261	Hypermethylation	Pancreatic cancer	C-myc	Repressing c-Myc expression	Liu et al. (2020c)
BLAT1	Hypomethylation	Breast cancer		Increased apoptosis, accumulation of DNA damage	Han et al. (2018)
LINC00886	Hypermethylation	Laryngeal squamous cell carcinoma		Mitigated cell proliferation, migration and invasion, VEGFA/PI3K/AKT signaling pathways and epithelial-mesenchymal transition	Lan et al. (2020)
SSTR5-AS1	Hypermethylation	Laryngeal squamous cell carcinoma	E-cadherin	Inhibits laryngeal carcinoma cells proliferation, migration and invasion	Wang et al. (2019a)
GAS5	Hypermethylation	Cervical cancer		Inhibited proliferation, cell cycle progression, invasion, migration while inducing apoptosis	Yang et al. (2019b)
MALAT1	Hypomethylation	Non-small cell lung cancer	CXCL5	Decrease cell migration and invasion	Guo et al. (2015)
TRPM2-AS1	Hypomethylation	Colorectal cancer		Promote proliferation and drug resistance of colorectal cancer cell	Ghasemi et al. (2021)

In addition to promoter hypermethylation, hypomethylation is also widely observed for many lncRNA genes. For instance, the well-known lncRNA *H19* displays aberrant promoter hypomethylation in many different cancer-types, including bladder cancer (Takai et al., 2001), colorectal cancer (Tian et al., 2012), and oral squamous cell carcinoma (Lee et al., 2021). One exception was found in the peripheral blood of gastric cancer patients, where hypermethylation of *H19* was observed that associated with poor prognosis (Hu et al., 2021). Another lncRNA *PlncRNA-1* was found hypomethylated in breast cancer tissue and accompanied by overexpression. It also functions as a ceRNA in the regulatory axis of miR-136—Smad3, regulating epithelial–mesenchymal transition (EMT) (Kang et al., 2020). Besides proximal promoter regions, aberrant DNA methylation at enhancer region has also been observed for lncRNA genes. For example, hypomethylation of the enhancer mapping to *Esrp2-as* is associated with its overexpression in breast cancer. This lncRNA locates in proximity to *Esrp2* (epithelial splicing regulatory protein 2), coordinated overexpression of *Esrp2* and *Esrp2-as* inversely correlates with hypomethylation in the enhancer and promotes cell motility and proliferation (Heilmann et al., 2017). Some other representative examples of aberrant methylation of lncRNA promoter in different cancers are summarized in Table 1.

In recent years, circRNA as another important class of non-coding RNAs has gained much attention due to its promising regulatory roles in cellular systems. CircRNAs are generated from precursor mRNA and are derived from non-canonical back-splice junction by linking 3′ splice site to a downstream 5′ splice site (Ashwal-Fluss et al., 2014). In this case, circRNA are thought to share the same transcription regulatory mechanism with their host genes. A previous study found a group of six circRNAs with their host genes undergo cancer-specific hypermethylation-associated transcriptional silencing, this phenomenon is suggested to be wide spread among different types of human malignancies (Ferreira et al., 2018). Another example was from multiple myeloma (MM), circRNA *ciRS-7* is downregulated in MM cells with immunomodulatory drug resistance. The decrease of its expression is associated with promoter hypermethylation of its host gene *LINC00632* (Jakobsen et al., 2021). However, evidence also suggests that many circRNAs may be transcriptionally regulated independently from their linear isoforms, resulting in different levels between their expression and that of their cognate linear mRNAs (Salzman et al., 2013; Rybak-Wolf et al., 2015). But the detailed mechanism of epigenetic regulation on circRNA biogenesis is largely unknown and remains further investigation.

It is worth noting that improvements in high-throughput sequencing technologies have led to the development of DNA methylome approaches, such as Whole Genome Bisulfite Sequencing (WGBS), Reduced Representation Bisulfite Sequencing (RRBS), DNA Immunoprecipitation Sequencing (MeDIP-seq), Methylation-sensitive restriction enzyme digestion sequencing (MRE-seq) and Human Methylation BeadChip Array (450K, EPIC). These technologies allow comprehensive characterization of human cancers *via* integrative analyses of genome, epigenome, and transcriptome data, and enable identification of global aberrant epigenetic patterns implicating deregulated lncRNAs and circRNAs. For example, by applying a combined strategy of MeDIP-seq and MRE-seq, Zhang et al. (2014) investigated the genome-wide DNA methylome profile in endometrial cancer, with hundreds of differentially methylated regions (DMRs) identified that co-localized with the promoters of lncRNA genes, including the well-known *Xist* which is critical for establishing inactivation of the X chromosome. Another study based on integrative analysis of MeDIP-seq and RNA-seq data identified differentially methylated lncRNAs in bladder cancer, with 26 lncRNAs presenting reverse correlation between methylation and expression (Zhang et al., 2019). Another integrative analysis of RRBS and RNA-seq, now in lung cancer, identified eight lncRNAs whose expression are associated with methylation in promoter regions (Sun et al., 2021). Due to the complex processing procedures and high cost of high-throughput sequencing based methylome technology, studies that identify global DNA methylation patterns for lncRNAs are still limited. For this reason, the Illumina Infinium Human Methylation450 BeadChip Array and its successor, the MethylationEPIC Array, are now commonly used to investigate DNA methylation profiles for different scenarios. Many studies have developed re-annotation strategies to identify array probes located in genome loci that associated with lncRNAs and to obtain lncRNA methylation profiles for a large number of samples (Zhi et al., 2014; Zhi et al., 2018). For example, one study performed in-depth characterization of DNA methylation landscape of lncRNA genes in 20 cancer types from The Cancer Genome Atlas (TCGA), discovering that the expression of lncRNAs is recurrently activated in tumors by hypomethylation. Overexpression of lncRNA *EPIC1* was identified to enhance tumor growth *in vitro* and *in vivo* for breast cancer, and is associated with poor prognosis of the patients (Wang et al., 2018b). Many other studies utilized bioinformatics and systems biology approaches to investigate differential methylation patterns of lncRNAs and their associated functions at pan-cancer wide (Ma et al., 2017; Xiao et al., 2018; Li et al., 2020; Ji et al., 2020; Xu et al., 2021; Zhong et al., 2021; Zhao et al., 2022). Although most of these DNA methylation related lncRNA dysregulation remains further confirmation and mechanism investigation, these current progresses indicate that many lncRNA genes are recurrently targeted by DNA methylation alterations in tumors, and could play an important role in tumor initiation and progression, and are worth being further evaluated for usage as cancer biomarkers.

## Long non-coding RNAs as DNA methylation regulator

One of the major advances for functional study of lncRNAs over the past decade has been their participation in epigenetic control. The regulation by lncRNAs on DNA methylation has been proved to be an important mechanism that controls gene expression during cancer development (Ferreira and Esteller, 2018). For instance, we have previously shown that the well-known lncRNA *HOTAIR* is associated with methylation profile enriched for polycomb group target (PCGT) genes in ovarian cancer, this *HOTAIR*-associated DNA methylation signature could serve as biomarkers for mesenchymal differentiation and also as for carboplatin resistance of the tumor cell (Teschendorff et al., 2015). LncRNA associated DNA methylome deviation is achieved through direct or indirect interactions with DNMT or TET members to recruit or sequester these enzymes from specific genome loci, resulting in promotion or repression of the DNA methylation in *cis* or in *trans*. *HOTAIR* and some other lncRNAs, such as particle, are found to recruit epigenetic modifiers to RNA binding loci in the genome by formation of triple helix, which functions to modulate global methylation in cancer cells (Kalwa et al., 2016; O'Leary et al., 2017). The effect of lncRNAs on DNA methylation dysregulation of their target genes affects multiple cellular regulatory networks, revealing their importance for tumorigenesis and progression.

### Long non-coding RNAs interact with DNA methyltransferases

**FIGURE 2:**
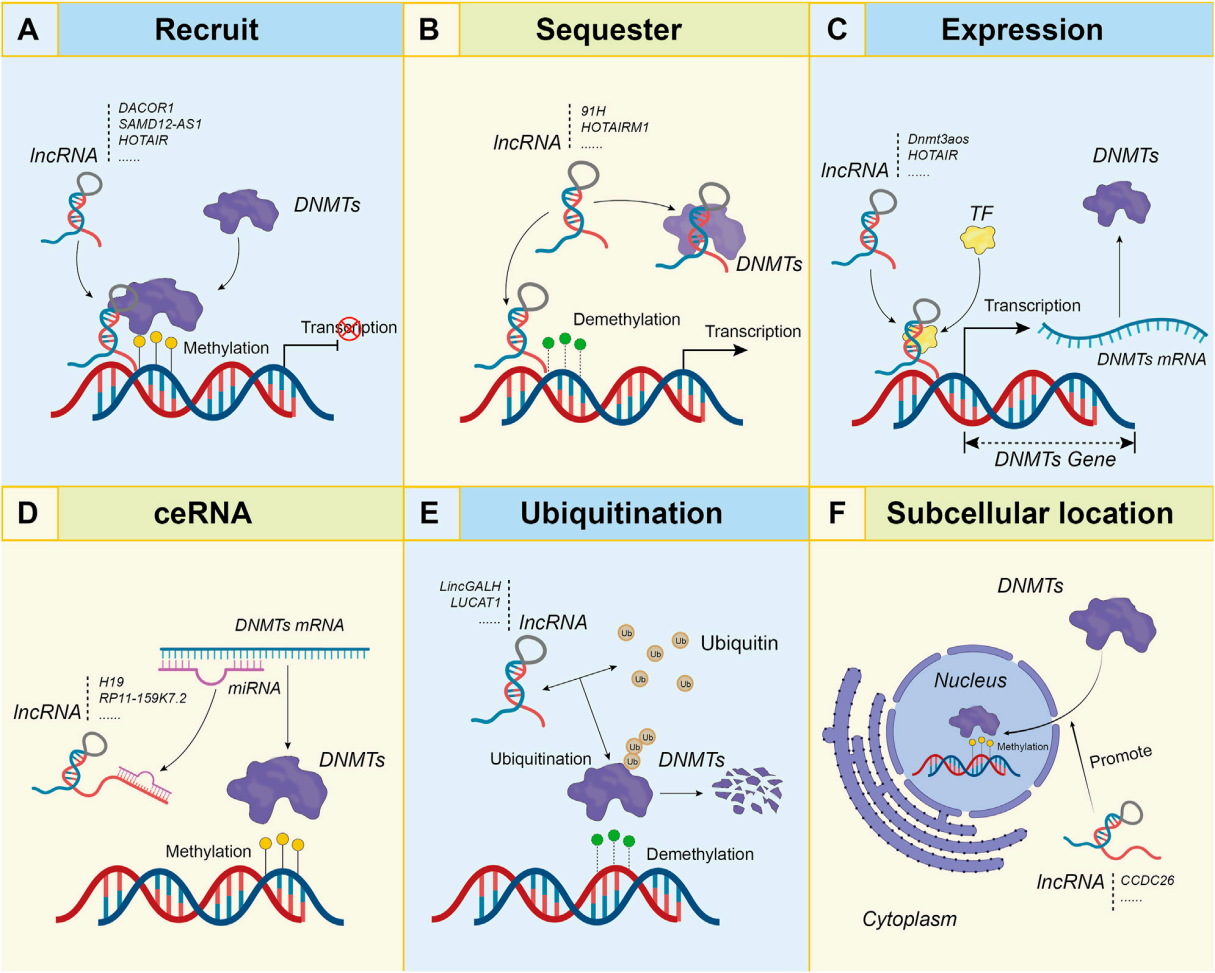
Detailed mechanism for DNA methylation regulation by lncRNAs in direct mode. **(A)**. LncRNAs recruit DNMTs to genome loci; **(B)**. LncRNAs sequester DNMTs from genome loci; **(C)**. LncRNAs regulate expression level of DNMTs; **(D)**. LncRNAs function as ceRNA to regulate DNMT expression level; **(E)**. LncRNAs influence the ubiquitination of DNMT proteins to affect the degradation. **(F)**. LncRNAs promote subcellular location of DNMT proteins. It is worth noting that similar mechanisms also applies to TET family members.

As the core enzyme involved in DNA methylation, interfering with DNMTs could be the most effective way for its function disturbance. Many lncRNAs were identified that physically interact with DNMTs to regulate methylation on target genes (Figure 2A). Merry et al. (2015) discovered 148 lncRNAs that interact with DNMT1 in colon cancer by using the RNA immunoprecipitation sequencing (RIP-seq) method. Among these, one named *DACOR1* (DNMT1-associated colon cancer repressed lncRNA 1), which presents downregulated expression in colon cancer, was identified to interact with DNMT1 and recruit this macromolecular complex at specific genomic sites to influence DNA methylation and gene expression. Induction of *DACOR1* in colon cancer cells results in global hypermethylation at multiple loci without changing the *DNMT1* expression level, many of the hypermethylated regions are associated with genes that participate in cancer related pathways, such as TGF-β/BMP signaling (Somasundaram et al., 2018). Similarly, another lncRNA *SAMD12-AS1* was found highly up-regulated in gastric cancer. *SAMD12-AS1* may facilitate the repression of *p53* by recruiting DNMT1, thus promoting the progression of gastric cancer (Lu et al., 2021). In chronic myelocytic leukemia (CML), the lncRNA *HOTAIR* was found to enhance the methylation of *PTEN* promoter by recruiting DNMT1. Overexpression of *HOTAIR* could facilitate the proliferation, invasion, and migration of CML cells (Song et al., 2021). Besides PCGs, lncRNAs associated DNA methylation dysregulation are also widely found in promoters of other types of ncRNAs, such as miRNA. In hepatocellular carcinoma (HCC), miR-122 was identified as the methylation target of *HOTAIR*, the downregulated expression of miR-122 by *HOTAIR* leads to the activation of oncogene *Cyclin G1* and promotion of tumorigenesis in HCC (Cheng et al., 2018). Another example is *TINCR*, this lncRNA can recruit DNMT1 to the promoter of miR-503 gene in breast cancer. Overexpression of *TINCR* could increase methylation and suppress the transcription of miR-503-5p. Of note, *TINCR* can also act as a ceRNA for miR-503-5p to regulate *EGFR* and interfere with JAK2–STAT3 signaling (Wang et al., 2021).

Besides the recruitment mechanism, lncRNA also sequester DNMTs from particular genome loci by a competitive interaction mode (Figure 2B). A lncRNA arising from the *CEBPA* gene locus termed *ecCEBPA* could compete with DNMT1, thus inhibit methylation of *CEBPA* gene and facilitate *CEBPA* expression in leukemic cells. (Di Ruscio et al., 2013). This lncRNA was later identified to interact with DNA strand by forming a DNA:RNA triple helices and protect regions near its binding site from methylation (Ogunleye et al., 2021). Another lncRNA, named *91H* which located at the *H19*/*IGF2* locus and transcribed in *H19* antisense orientation, is overexpressed in breast cancer and prevent the maternal allele at the *H19*/*IGF2* locus from DNA methylation, by this mechanism to induce overexpression of oncogenic *H19* (Vennin et al., 2017). LncRNA *HOTAIRM1* (HOX antisense intergenic RNA myeloid 1), which is located between the *HOXA1* and *HOXA2* genes, could interact with DNMTs and other epigenetic factors to sequester them away from *HOXA1* promoter in glioblastoma multiforme (GBM). Upregulation of *HOTAIRM1* could lead to reduced methylation levels of *HOXA1* and finally to its upregulation of expression (Li et al., 2018). A similar observation was found in dental follicle stem cells (hDFSCs), in which *HOTAIRM1* binding to the CpG islands of the *HOXA2* promoter and reduce the binding of DNMT1 at the *HOXA2* promoter, resulting in *HOXA2* hypomethylation and deviant induction (Chen et al., 2020). These examples indicate that this regulatory mechanism by *HOTARIM1* within the *HOXA* cluster could be universal across tissues and diseases.

LncRNAs are also found to interact with other DNA methyltransferases in addition to DNMT1 to influence the methylation pattern of target genes. For instance, lncRNA *HOTAIR* was shown to recruit DNMT3B to increase *HOXA5* promoter methylation and silence its expression in acute myeloid leukemia (AML). *HOTAIR* silence and *HOXA5* activation were found to induce apoptosis and reduce proliferation of AML cells (Wang et al., 2019d). Another lncRNA *MROS-1* was found to modulate tumor suppressor *PRUNE2* expression by interacting with DNMT3A in oral squamous cell carcinoma (OSCA). Higher methylation levels of *PRUNE2* promoter induced by *MROS-1* were associated with cell migration and metastases (Su et al., 2021). The lncRNA *TTTY15* could interact with DNMT3A and prevent its binding to *TBX4* promoter in non-small cell lung cancer (NSCLC), the lower expression level of *TTTY15* and the associated downregulation of *TBX4* is connected with metastasis and worse prognosis of NSCLC patients (Lai et al., 2019).

Besides interacting with DNMT proteins, lncRNAs could also regulate their expression level with different mechanisms (Figure 2C). For instance, one lncRNA named *Dnmt3aos* (DNA methyltransferase 3A, opposite strand) located on the antisense strand of *DNMT3A* was found to participate in the regulation of *DNMT3A* expression. *Dnmt3aos* is highly expressed in M(IL-4) macrophages, which leads to the highly coordinated expression of this sense-antisense pair of *DNMT3A* and *Dnmt3aos*. Elevated expression of *Dnmt3aos* and *DNMT3A* results in global DNA methylation changes in M(IL-4) macrophages (Li et al., 2020). In small cell lung cancer (SCLC), *HOTAIR* was found to inhibit expression of *DNMT1* and *DNMT3B*, thus regulating the methylation of *HOXA1* to mediate chemoresistance of SCLC (Fang et al., 2016). Whereas in AML patients, *HOTAIR* present up-regulated expression, which leads to downregulation of *PTEN via DNMT3B*-dependent pathway, and lead to doxorubicin resistance (Zhou et al., 2021).

LncRNAs have long been recognized to regulate gene expression *via* the ceRNA mechanism, by which lncRNAs act as a “sponge” to combine with miRNAs and sequester their interactions with mRNAs to de-repress the expression of targets. Many examples have been found for lncRNAs that regulate the expression of DNMTs as ceRNA (Figure 2D). In laryngeal squamous cell carcinoma (LSCC), *H19* was found to be the sponge for miR-148a-3p, through which to regulate *DNMT1* expression. Overexpression of *H19* in LSCC leads to elevated expression of *DNMT1* and genome wide change of DNA methylation, including *MGMT* (Wu et al., 2016). Similar observation was also found for the *RP11-159K7.2*—miR-206 – *DNMT3A* axis in LSCC. Overexpressed *RP11-159K7.2* could interact with miR-206, which binds with *DNMT3A* 3′-UTR. Interestingly, *DNMT3A* was also found to inhibit the expression of miR-206 *via* a DNA methylation-dependent manner, thus a feedback loop is maintained between *DNMT3A* and miR-206 to keep its internal balance (Wang et al., 2020). In hepatocytes, *HOTAIR* was found as sponge of miR-29b, which also regulates the expression of *DNMT3B* to regulate the methylation level of *PTEN* (Yu et al., 2020). Besides interactions with miRNAs, lncRNAs are also found to regulate the mRNA level of DNMTs by interacting with other proteins. For instance, the *RMST*, a lncRNA capable of upregulating *DNMT3B* expression by interaction with the RNA binding protein HuR, leads to alterations in global methylation in cancers (Peng et al., 2020).

LncRNAs could also function to regulate protein expression for DNMTs, such as by mechanism of ubiquitination (Figure 2E). In HCC, lncRNA *linc-GALH* overexpression could enhance the ubiquitination of DNMT1 to accelerate its degradation. In this way, *linc-GALH* reduces the methylation level of *Gankyrin* to promote its expression (Xu et al., 2019). In another example, lncRNA *LUCAT1* was found to interact with DNMT1 but now to inhibit the ubiquitination in esophageal squamous cell carcinoma (ESCC). Upregulated *LUCAT1* thus stabilizes DNMT1 to enhance the methylation and inhibit the expression of tumor suppressors (Yoon et al., 2018). In addition, lncRNAs could also regulate local concentration of DNMTs by interfering with its subcellular location (Figure 2F). For example, the lncRNA *CCDC26* could promote DNMT1 localization from cytoplasm to nucleus. In absence of *CCDC26*, DNMT1 is found mis-located in the cytoplasm, resulting in global hypomethylation (Jones et al., 2021). Examples of lncRNAs that interact with DNMTs to regulate methylation of downstream genes and their functions in cancers are summarized in Table 2.

**TABLE 2:** Representative lncRNAs that regulate DNA methylation of other genes in cancers and other disease.

LncRNA name	Cofactor	Interaction mode	Target	Tissue/cancer	Function	References
DACOR1	DNMT1	Recruit	Genome wide	Colon cancer	TGF-β/BMP signaling	Merry et al. (2015); Somasundaram et al. (2018)
SAMD12-AS1	DNMT1	Recruit	p53	Gastric cancer	P53 signaling pathway	Lu et al. (2021)
HOTAIR	DNMT1	Recruit	PTEN	Chronic myelocytic leukemia		Song et al. (2021)
HOTAIR	DNMT1	Recruit	miR-122	Hepatocellular carcinoma	Cyclin G1 repression	Cheng et al. (2018)
TINCR	DNMT1	Recruit	miR-503-5p	Breast cancer	EGFR and JAK2–STAT3 signaling	Wang et al. (2021b)
ecCEBPA	DNMT1	Sequester	CEBPA; genome wide			Di Ruscio et al. (2013); Ogunleye et al. (2021)
91H	DNMT1	Sequester	H19; IGF2	Breast cancer		Vennin et al. (2017)
HOTAIRM1	DNMTs; G9a; EZH2	Sequester	HOXA1	Glioblastoma multiforme		Li et al. (2018)
HOTAIRM1	DNMT1	Sequester	HOXA2	Dental follicle stem cell	Osteogenesis	Chen et al. (2020b)
HOTAIR	DNMT3B	Recruit	HOXA5	Acute myeloid leukemia	Apoptosis	Wang et al. (2019d)
MROS-1	DNMT3A	Recruit	PRUNE2			Su et al. (2021)
TTTY15	DNMT3A	Sequester	TBX4	Non-small cell lung cancer	Metastasis	Lai et al. (2019)
Dnmt3aos	DNMT3A	Expression	Genome wide	M(IL-4) macrophage	Macrophage polarization	Li et al. (2020a)
HOTAIR	DNMT1; DNMT3B	Expression	HOXA1	Small cell lung cancer	Chemoresistance	Fang et al. (2016)
HOTAIR	DNMT3B	Expression	PTEN	Acute myeloid leukemia	Adriacin doxorubicin resistance	Zhou et al. (2021b)
H19	miR-148a-3p—DNMT1	ceRNA	MGMT; Genome wide	Laryngeal squamous cell carcinoma	Cell proliferation	Wu et al. (2016)
RP11-159K7.2	miR-206—DNMT3B	ceRNA	miR-206	Laryngeal squamous cell carcinoma		Wang et al. (2020)
HOTAIR	miR-29b—DNMT3B	ceRNA	PTEN	Hepatocytes	Liver fibrosis	Yu et al. (2020a)
RMST	HuR—DNMT3B	RNA stability	Genome wide			Peng et al. (2020)
Linc-GALH	Ubiquitin—DNMT1	Ubiquitination	Gankyrin	Hepatocellular carcinoma	AKT signaling	Xu et al. (2019c)
LUCAT1	Ubiquitin—DNMT1	Ubiquitination		Esophageal squamous cell carcinoma	Cell proliferation, apoptosis	Yoon et al. (2018)
CCDC26	DNMT1	Subcellular location	Genome wide		Apoptosis	Jones et al. (2021)
MAGI2-AS3	TET1	Recruit	MAGI2	Breast cancer	Cell proliferation and migration	Xu et al. (2021b)
MAGI2-AS3	TET2	Recruit	LRIG1	Acute myeloid leukaemia	Leukaemic stem cell self-renewal suppression	Chen et al. (2020a)
TARID	GADD45A—TET1	Recruit	TCF21			Arab et al. (2014); Arab et al. (2019)
HOTAIR	TET1	Expression	SOX17; MAGI2	Cervical cancer (Hela cell)	Wnt/β-catenin signaling	Salmeron-Barcenas et al. (2019)
H19	let-7—TET1	ceRNA	TGFBR2; TSP1	Atherosclerotic coronary arteries	TGF-β signaling	Cao et al. (2020)
H19	let-7—TET3	ceRNA	HMGA2	Uterine leiomyomas	Proliferation	Cao et al. (2019)
TETILA	TET2	Ubiquitination; subcellular location; recruit	MMP-9	Diabetic skin	Wound healing	Zhou et al. (2019a)
PYCARD-AS1	G9a; DNMT1	Recruit	PYCARD	Breast cancer		Miao et al. (2019)
KCNQ1OT1	HP1α	Recruit	Genome wide	Lung fibroblast	Heterochromatin reorganization	Zhang et al. (2022b)
LINC01133	EZH2	Recruit	DKK1	Pancreatic cancer	Wnt signaling	Weng et al. (2019)
HOXB13-AS1	EZH2; DNMT3B	Recruit	HOXB13	Glioma		Xiong et al. (2018)
Lnc-LALC	EZH2; DNMTs	Recruit	LZTS1	Colorectal cancer	Liver metastasis	Zhang et al. (2021a)
LUCAT1	EZH2; DNMTs	Recruit	CXXC4; SFRP2	Gastric cancer	Wnt/β-catenin signaling	Byun et al. (2020)
SNHG22	EZH2; DNMT1	Recruit	miR-16-5p	Hepatocellular carcinoma	Cell proliferation	Zhang et al. (2021c)
GIHCG	EZH2; DNMT1	Recruit	miR-200b/a/429	Hepatocellular carcinoma	Cell proliferation and migration	Sui et al. (2016)
SChLAP1	EZH2; DNMT3A; miR-340-5p—DNMT3A	Recruit; expression	miR-340-5p; miR-143-3p; miR-145-5p	Prostate cancer	Cell proliferation and migration	Huang and Tang, (2021)
HOXA11-AS	EZH2; LSD1; DNMT1; miR-1297—EZH2	Recruit; ceRNA	PRSS8; KLF2	Gastric cancer	Cell proliferation, migration and apoptosis	Sun et al. (2016)
LINC00470	miR-101—EZH2; miR-101—EED	ceRNA	ELFN2	Glioblastoma	Cell autophagy	Liu et al. (2018)
H19	SAHH	Interaction	Nctc1; genome wide			Zhou et al. (2015)
H19	SAHH	Interaction	HNF4α	Liver of metformin-exposed fetuses	Liver development and function	Deng et al. (2017)
H19	SAHH	Interaction	Beclin1	Breast cancer	Autophagy	Wang et al. (2019c)
H19	SAHH	Interaction	LINE-1	Lung		Fu et al. (2018)
SNHG6	miR-1297—MAT2A; MAT1A	ceRNA; subcellular location	Genome wide	Hepatocellular carcinoma		Guo et al. (2018)
LINC00662	MAT1A; SAHH	Interaction	Genome wide	Hepatocellular carcinoma		Guo et al. (2020)
PARTICLE	G9a; SUZ12	Recruit	MAT2A	Breast cancer cell line	Response to irradiation	O'Leary et al. (2015)
LINC00261	DNMTs	Recruit	DYPD	Esophageal cancer	5-fluorouracil resistance	Lin et al. (2019)
LINC01419	DNMTs	Recruit	GSTP1	Esophageal cancer	5-fluorouracil resistance	Chen et al. (2019b)
LINC00673	DNMTs	Recruit	KLF4	Prostate cancer	Paclitaxel resistance	Jiang et al. (2020)
LINC00628	DNMTs	Recruit	LAMA3	Lung adenocarcinoma	Vincristine resistance	Xu et al. (2019b)
LINC00607	DNMTs	Recruit	CASP9	Thyroid cancer	Doxorubicin resistance	Li et al. (2021a)
91H	DNMTs	Recruit	CDK4	Osteosarcoma	Tumor migration and invasion	Cheng et al. (2021)
H19	DNMT3B	Expression	Genome wide	Endometrial cancer; breast cancer	Cell proliferation	Zhong et al. (2017)
HOTAIR	EZH2; DNMTs	Interaction	ALDH1A1	Ovarian cancer	Spheroid formation and colony-forming	Wang et al. (2021c)
HOTAIR	miR-126—DNMT1	ceRNA	CDKN2A	Osteosarcoma	Cell viability and apoptosis	Li et al. (2017)
LINC00240	miR-124-3p—DNMT3B	ceRNA	miR-124-3p	Gastric cancer	Cell proliferation, invasion and migration	Li et al. (2020c)
XIST	miR-149-5p—DNMT3A	ceRNA	miR-149-5p	Cartilage	Cell proliferation, apoptotic and ENC degradation	Liu et al. (2020d)
HOTTIP	miR-101—DNMT3B	ceRNA	HoxA13	Cartilage	Cartilage development and destruction	Kim et al. (2013)
IRAIN	DNMT1; DNMT3A; DNMT3B	Recruit	VEGFA	Renal carcinoma	Cell proliferation, migration and apoptosis	Li et al. (2020b)
AS1DHRS4	G9a; EZH2	Recruit	DHRS4L1; DHRS4L2			Li et al. (2012)
PRKCA-AS1	DNMT1	Recruit	PRKCA	Heart	p38/MAPK pathway	Xie et al. (2021)
LINC00518	DNMT1; DNMT3A; DNMT3B	Recruit	CDX2	Breast cancer	Cell proliferation, invasion, migration and EMT	Wang et al. (2019b)
RCPCD	DNMT1; DNMT2; DNMT3	Recruit	HCN4	Embryonic stem cells	Differentiation of ESCs into pacemakelike cells	Zhu et al. (2021)
LINC00313	DNMT1; DNMT3B	Recruit	ALX4	Thyroid cancer	AKT/mTOR signaling, cell proliferative, migratory, invasive abilities as well as EMT	Zhao and Hu, (2019)
LINC00152	DNMTs	Recruit	BRCA1/PTEN	Breast cancer	Tumorigenesis and metastasis	Wu et al. (2018)
LINC00470	DNMT3A	Recruit	PTEN	Endometrial cancer	Cell invasiveness, migration and angiogenesis, facilitate tumorigenesis and metastasis	Yi et al. (2021)
LINC00922	DNMT1; DNMT3A; DNMT3B	Recruit	NKD2	Breast cancer	Wnt signaling pathway	Wang et al. (2021d)
LINC01419	DNMT1; DNMT3A; DNMT3B	Recruit	ZIC1	Hepatocellular carcinoma	PI3K/Akt signaling pathway, tumor formation and metastasis	Hou et al. (2021)
MIR210HG	DNMT1	Recruit	CACNA2D2	Non-small cell lung cancer	Cell proliferation and migration	Kang et al. (2019)
SNHG1	DNMT1	Recruit	Bcl-2	Sepsis	Cell inflammation and apoptotic	Zhang et al. (2022a)
ADAMTS9-AS2	DNMT1; DNMT3A; DNMT3B	Recruit	CDH3	Esophageal cancer	Cell proliferation, invasion and migration	Liu et al. (2020a)
ELFN1-AS1	DNMT1; DNMT3A; DNMT3B	Recruit	ZBTB16	Gastric cancer	PI3K/AKT signaling pathway	Zhuang et al. (2022)
IGF2-AS	DNMT1	Recruit	IGF2	Breast cancer	PI3K/AKT/mTOR signaling pathway	Zhang et al. (2021d)
NEAT1	G9a; DNMT1; Snail	Recruit	CDH1	Osteosarcoma	Metastasis *in vitro* and *in vivo*, EMT	Li and Cheng, (2018)
PCAT-14	DNMT1; DNMT3A; DNMT3B	Recruit	miR-372	Hepatocellular carcinoma	Cell proliferation, invasion, cell cycle arrest	Wang et al. (2017)
HAGLR	DNMT1	Recruit	E2F1	Lung adenocarcinoma	Cell growth	Guo et al. (2019)
XIST	DNMT1; DNMT3A; DNMT3B	Recruit	TIMP-3	Cartilage	Collagen degradation	Chen et al. (2019a)
RAMP2-AS1	DNMT1; DNMT3B	Recruit	CXCL11	Breast cancer	Tumor growth	Li et al. (2022a)
TNRC6C-AS1	DNMT1; DNMT3A; DNMT3B	Recruit	STK4	Thyroid cancer	Hippo signaling pathway	Yang et al. (2019a)
yylncT	DNMT3B	Recruit		Embryo	Embryonic cell fate transition	Frank et al. (2019)
SNHG1	DNMT1	Expression	PTBP1	Bone marrow	Adipogenic differentiation and contributed to osteoporosis	Yu et al. (2022a)
FAS-AS1	DNMT3B	Expression	SIRT1; FAS	Leukemia		Yuan et al. (2020)
Linc-POU3F3	EZH2; DNMT1; DNMT3A; DNMT3B	Recruit	POU3F3	Esophageal squamous cell carcinoma	Cell proliferation and ability to form colonies	Li et al. (2014)
PVT1	EZH2; DNMT1	Recruit	miR-18b-5p; HIF1A	Gallbladder cancer	Cell proliferation	Jin et al. (2020)
ROIT	DNMT3A	Ubiquitination	Nkx6.1	Pancreas islet	Glucose homeostasis and insulin transcription	Zhang et al. (2020a)
Platr10	TET1	Recruit	Oct4		Modulating chromatin architecture	Du et al. (2021b)
WT1-AS	TET2; TET3; DNMTs	Recruit	WT1	Leukemia		McCarty and Loeb, (2015)
NEAT1	DNMTs	Recruit	miR-129-5p; WNT4	Breast cancer	WNT signaling	Lo et al. (2016)
Evf2	MECP2	Recruit	DLX1/2	Forebrain		Berghoff et al. (2013)
NKILA	NF-κB; DNMT3A	Recruit	KLF4	Vascular endothelium	Endothelium inflammation	Zhu et al. (2019)
HOTAIR	DNMT1; DNMT3B; EZH2	Expression	HOXA1	Small cell lung cancer	Multidrug resistance	Fang et al. (2018)
ANRIL	EZH2	Recruit	ERRFI1	Cholangiocarcinoma	Cell proliferation and migration	Yu et al. (2020b)
LINC00858	DNMTs	Recruit	WNK2	Colon cancer	Cell apoptosis, autophagy and senescence	Wu et al. (2020)
UCA1	EZH2	Recruit	p21	Breast cancer	PI3K/AKT signaling pathway	Li et al. (2019)
H19	EZH2	Recruit	BIK	Breast cancer	Paclitaxel (PTX) resistance	Si et al. (2016)
AC092723.1	TET1	Recruit	IRF8			Zhou et al. (2022)
HOTAIR	EZH2	Recruit	E-cadherin	Oral squamous cell carcinoma	Cell invasion, migration and apoptosis	Wu et al. (2015)
LINC00887	DNMT1	Recruit	CA9	Tongue squamous carcinoma	Suppress oncogenic CA9	Shen et al. (2021b)
LINC00472	DNMTs	Recruit	MCM6	Breast cancer	Inhibite tumor growth and metastasis	Shao et al. (2021)
LINC01270	DNMTs	Recruit	GSTP1	Esophageal cancer	Cell proliferation, migration, invasion and drug resistance	Li et al. (2021b)
HOTAIR	DNMTs	Recruit	MTHFR	Esophageal cancer	Cell apoptosis and proliferation	Zhang et al. (2020b)
BZRAP1-AS1	DNMT3B	Recruit	THBS1	Hepatocellular carcinoma	Angiogenesis and tumor growth	Wang et al. (2019e)
PVT1	DNMT1	Recruit	BNIP3	Gastric cancer	Cell proliferation	Xin et al. (2021)
SNHG3	EZH2	Recruit	MED18	Gastric cancer	Cell migration and invasion	Xuan and Wang, (2019)
HOTAIR	DNMT1; EZH2	Recruit	miR-454-3p	Gastric cancer	Cell apoptosis and autophagy	Bao et al. (2017)
LINC00630	DNMT3B; EZH2	Recruit	BEX1	Colorectal cancer	Cell apoptosis and radio-resistance	Liu et al. (2020b)
Lnc34a	DNMT3A; PHB2	Recruit	miR-34a	Colorectal cancer	Cell proliferation	Wang et al. (2016)
SATB2-AS1	TETs; GADD45A	Recruit	SATB2	Colorectal cancer	Cell metastasis and immune response	Xu et al. (2019a)
Dali	DNMT1	Recruit	Pou3f3; genome wide	Central nervous system	Cell differentiation	Chalei et al. (2014)
Dum	DNMT1; DNMT3A; DNMT3B	Recruit	Dppa2	Skeletal myoblast cell	Myogenesis	Wang et al. (2015)
lincRNA-p21	HNRNPK – DNMT1; SETDB1	Recruit	Nanog	Pluripotent stem cell	Cell differentiation	Bao et al. (2015)
Kcnq1ot1	DNMT1; EZH2; G9a	Recruit	Kcnq1	Placenta	Gene imprinting	Mohammad et al. (2010)
THAP9-AS1	DNMTs	Recruit	SOCS3	Osteosarcoma	JAK2/STAT3 signaling	Yang et al. (2021b)
H19	PRC2	Recruit	genome wide	Neuroendocrine prostate cancer	Metastatic	Singh et al. (2021)
KCNQ1OT1	DNMT1	Recruit	PTEN	Triple negative breast cancer	Cell proliferation, invasion, and migration	Shen et al. (2021a)
KCNQ1OT1	DNMT1; DNMT3A; DNMT3B	Recruit	EIF2B5	Ovarian cancer	Metastasis	He et al. (2022)
KAT7	DNMTs	Recruit	miR-10a	Non-small cell lung cancer		Gao et al. (2021)
SNHG3	miR-448—DNMT1	ceRNA	SEPT9	Gastric cancer	Cell growth, metastasis	Li et al. (2022d)
KIF9-AS1	DNMT1	Recruit	RAI2	Hepatocellular carcinoma	Cell proliferation, migration, apoptosis	Yu et al. (2022b)
PVT1	EZH2; DNMT1	Recruit	ZBP1	Liver cell	Response to nonylphenol	Qiannan et al. (2022)
ZFAS1	DNMT3B	Recruit	Notch1	Myocardial ischemia-reperfusion injury	Apoptosis	Li et al. (2022b)
UCA1	EZH2; DNMT1	Recruit	APAF1	Myocardial ischemia-reperfusion injury		Jin et al. (2022)
LINC01270	DNMT1; DNMT3A; DNMT3B	Recruit	LAMA2	Breast cancer	MAPK signaling pathway	Li et al. (2022c)
UCA1	DNMT1; DNMT3A; DNMT3B	Recruit	METTL14	Breast cancer	Cell proliferation, invasion, metastasis	Zhao et al. (2022a)

### Long non-coding RNAs interact with ten-eleven translocation enzymes

DNMTs are responsible for catalyzing the conversion of cytosine to 5-mC whereas TET enzymes catalyze the successive conversion of 5mC to 5-hydroxymethylcytosine (5hmC), 5-formylcytosine (5fC), and 5-carboxylcytosine (5caC) to promote locus-specific removal of methylation. DNA demethylation can be achieved either as a process in the absence of functional DNA methylation maintenance mechanism during DNA replication or through TET-mediated 5mC oxidation. In this case, regulation to TET family affects the methylation level of downstream genes as well. Studies have discovered many lncRNAs interact with TETs to regulate methylation process (Table 2). For example, lncRNA *MAGI2-AS3* (MAGI2 antisense RNA 3) which is transcribed from the antisense strand near the *MAGI2*, acts as cis-acting factor to downregulate the DNA methylation level of the *MAGI2* promoter by interaction with TET1 and promotes apoptosis by activating the Fas/FasL signaling pathway in breast cancer (Xu et al., 2021). In AML, *MAGI2-AS3* recruits TET2 to the *LRIG1* promoter region in *trans* and causes DNA demethylation of *LRIG1*. Downregulation of *MAGI2-AS3* suppresses the self-renewal capacity of leukemic stem cell by promoting *LRIG1* expression (Chen et al., 2020). LncRNAs are also found to recruit TET enzymes in an indirect mode. The lncRNA *TARID* (TCF21 antisense RNA inducing demethylation) could interact with both the *TCF21* promoter and GADD45A protein, whereas GADD45A in turn recruits TET1 to activate the expression of *TCF21* (Arab et al., 2014). The authors further show that *TARID* combine to *TCF21* promoter to form an R-loop of DNA–RNA hybrids, which is recognized by GADD45A and then triggers TET1-dependent DNA demethylation (Arab et al., 2019).

TETs are also found to be regulated by lncRNAs at the transcriptional, posttranscriptional, and protein expression levels. In cervical cancer, the *HOTAIR* could regulate *TET1* expression, which leads to promoter hypermethylation of Wnt/β-catenin signaling related genes. In Hela cells, upregulated *HOTAIR* leads to the decreased *TET1* expression, which is associated with the transcriptional activity of Wnt/β-catenin pathway genes, such as *PCDH10*, *SOX17*, *AJAP1*, and *MAGI2* (Salmeron-Barcenas et al., 2019). At the posttranscriptional level, *TET1* is found to be regulated by lncRNA *H19 via* miRNA let-7 with ceRNA mode, *TET1* expression alteration due to upregulation of *H19* promotes TGF-β signaling related endothelial–mesenchymal transition in endothelial cells of atherosclerotic coronary arteries (Cao et al., 2020). A similar observation was found for *TET3* in uterine leiomyomas, a *H19*—let-7—*TET3* axis was identified for methylation regulation of fibroid-promoting gene and to drive proliferation of leiomyoma cells (Cao et al., 2019). At the protein expression level, a multifunctional lncRNA *TETILA* was found in diabetic skin that play a key role in wound healing. Zhou et al. (2019a) indicated this lncRNA could regulate TET2 stability through the ubiquitin-proteasome pathway and also promote TET2 nuclear translocation. In addition, *TETILA* also acts as a scaffold to recruit thymine-DNA glycosylase (TDG), which simultaneously interacts with TET2 at the promoter of *MMP-9* for its demethylation and transcriptional activation.

### Long non-coding RNAs interact with other epigenetic factors

**FIGURE 3:**
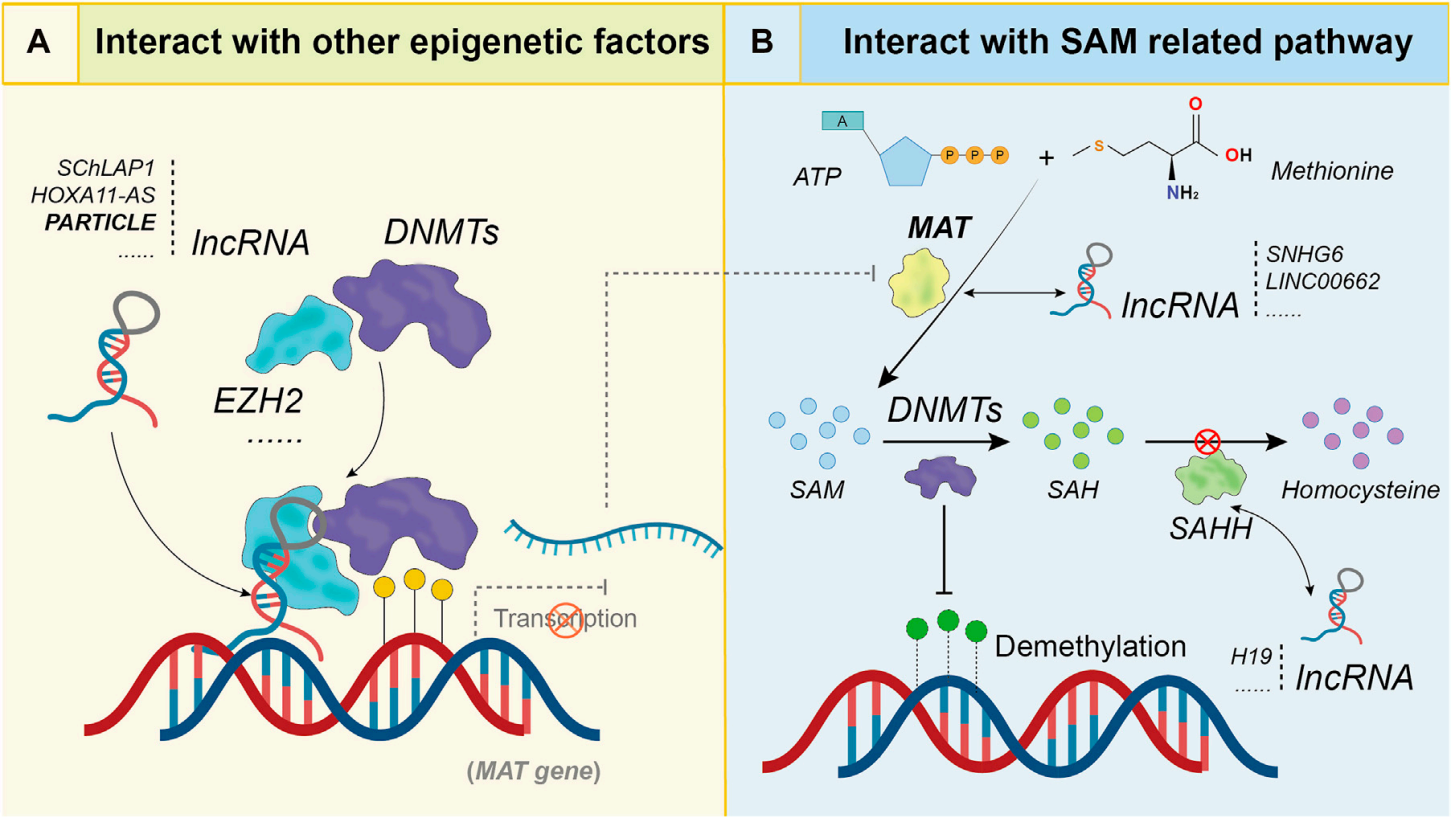
Detailed mechanism for DNA methylation regulation by lncRNAs in indirect mode. **(A)**. LncRNAs interact with other epigenetic factors, such as EZH2, to affect methylation level of downstream genes; **(B)**. LncRNAs interfere with DNMT functions by interacting with S-adenosylmethionine related pathway.

One of the most intriguing observations have recently emerged in epigenetics is the subtle crosstalk between DNA methylation and other epigenetic modifications. Accumulating literature has revealed complex mechanisms underlying the interplay between DNA methylation and histone modification. Many partners of DNMTs have been found that involved in both of the DNA methylation and histone modification. In addition, DNA methylation status within genome present concomitant presence with other repressive marks, such as histone deacetylation. For example, HDAC1 has the ability to bind DNMT1, the histone deacetylase activity is required for DNMT1 related DNA methylation maintenance in heterochromatin (Fuks et al., 2000). DNMTs have also been identified to interact with G9a, which is responsible for mono-, di-and slowly trimethylation of histone H3 lysine 9 (H3K9). This interaction has been shown to play a role in the establishment of DNA methylation pattern for key genes in ES cells (Xin et al., 2003; Esteve et al., 2006). In addition, the PRC2 system, which has histone methyltransferase activity for H3K27me3, is connected to DNA methylation related gene silencing at specific loci. The PRC2 core component EZH2-dependent recruitment of DNMT3A was found to be associated with H3K27me3 and DNA methylation (Jin et al., 2009; Rush et al., 2009; Li et al., 2021c). This explains how lncRNAs interact with epigenetic factors to regulate DNA methylation at particular loci (Figure 3A). For instance, the *PYCARD-AS1*, which is antisense to the pro-apoptotic gene *PYCARD*, functions to induce DNA methylation and H3K9me2 modification of *PYCARD* promoter by recruiting the chromatin-suppressor proteins G9a and DNMT1 in breast cancer (Miao et al., 2019). Another example is the lncRNA *KCNQ1OT1*, which binds and recruits the heterochromatin protein HP1α, and finally lead to DNA methylation and H3K9me3 modification in the genome. One repeat-rich region within *KCNQ1OT*1 is identified mainly responsible for Hoogsteen base pairing with double-stranded DNA, by which to fulfill the function of protein recruitment. This observation demonstrates an example for lncRNA to induce and maintain epigenetic silencing at repetitive DNA elements, in order to safeguard against genome instability (Zhang et al., 2022). In pancreatic cancer, the upregulated *LINC01133* was found to recruit EZH2 to for histone methylation and also to promote the promoter methylation of *DKK1*, thus activate Wnt signaling (Weng et al., 2019). LncRNA *HOXB13-AS1* is found upregulated in glioma and negatively correlated with its surrounding gene *HOXB13*, this lncRNA could increase DNMT3B-mediated methylation of *HOXB13* promoter by binding with EZH2 (Xiong et al., 2018). Similar examples include the regulation of *LZTS1* by *lnc-LALC* during liver metastasis of colorectal cancer (Zhang et al., 2021), regulation of *CXXC4* and *SFRP2* by *LUCAT1* in gastric cancer (Byun et al., 2020). In addition, lncRNA could also regulate promoter methylation of miRNA genes by interacting EZH2. For instance, lncRNA *SNHG22* was found to recruit DNMT1 to miR-16-5p DNA promoter through EZH2 and inhibited miR-16-5p transcription *via* DNA methylation (Zhang et al., 2021c). LncRNA *GIHCG* physically associates with EZH2 and recruits EZH2 and DNMT1 to promoter regions of the miR-200b/a/429, which lead to changes of H3K27me3 and DNA methylation levels in the miR-200b/a/429 promoter, and dramatically silences their expression (Sui et al., 2016).

It is worth noting that many miRNAs regulated by lncRNA through promoter DNA methylation are also found to regulate the upstream lncRNAs or other epigenetic factors, by which a feedback loop formed to control the internal gene expression. For example, lncRNA *SChLAP1* was found to recruit EZH2 and DNMT3A to repress multiple miRNA expression in prostate cancer, including the miR-340-5p/miR-143-3p/miR-145-5p, these miRNAs in turn regulate *DNMT3A* expression (Huang and Tang, 2021). In gastric cancer, EZH2 along with the histone demethylase *LSD1* and *DNMT1* were recruit by the lncRNA *HOXA11-AS*, this lncRNA also acts as sponge for miR-1297, antagonizing its ability to repress *EZH2* protein translation (Sun et al., 2016). In glioblastoma, *LINC00470* could enhance the expression of *ELFN2* through adsorption of miR-101, and also affect the methylation level of ELFN2 by decreasing H3K27me3 occupancy (Liu et al., 2018). The above examples indicate that lncRNAs are able to control genes at the transcriptional level or post-transcriptional level through a variety of different mechanisms to achieve accurate regulation of expression levels for downstream target genes.

### Long non-coding RNAs interact with S-adenosylmethionine related pathway

All DNA methyltransferases are known to use S-adenosylmethionine (SAM) as the methyl donor and generate S-adenosylhomocysteine (SAH) as by-product. The methyl donor SAM is synthesized from ATP and methionine by the methionine adenosyltransferase (MAT) (Lu and Mato, 2012), whereas SAH could be eliminated by S-adenosylhomocysteine hydrolase (SAHH), SAH also acts as feedback inhibitor of DNMTs (Lyko, 2018). Regulation on the genes involved SAM synthesis or SAH degradation by lncRNAs may lead to malfunction of DNMTs to interference DNA methylation (Figure 3B). The *H19* for instance, could bind to SAHH and inhibits its function of SAH hydrolyzing, then give rise to genome-wide methylation alteration (Zhou et al., 2015). This mechanism was further observed in liver of metformin-exposed fetuses to induce hypomethylation and increased expression of *HNF4α* (Deng et al., 2017), and also in tamoxifen-resistant breast cancer to induce the upregulation of *Beclin1* (Wang et al., 2019c), as well as in human lung tissue to regulate the *LINE-1* methylation (Fu et al., 2018).

Interference to MAT may result in the alteration of the SAM concentration and disturbance of DNA methylation process. This has been confirmed by the interaction between lncRNA *SNHG6* and MAT family members of *MAT1A* and *MAT2A*. On one hand, *SNHG6* was found to upregulate *MAT2A* expression by act as sponge for miR-1297, on another hand, this lncRNA also downregulate *MAT1A* translation by suppressing the nucleus-cytoplasmic shuttling of *MAT1A* mRNA, thereby regulate genome wide methylation in hepatoma cells of HCC (Guo et al., 2018). Another lncRNA *LINC00662* was identified to induce decay of *MAT1A* mRNA and also the degradation of SAHH protein by ubiquitination mechanism, in this way to reduce SAM and enhance SAH levels, which finally leads to global hypomethylation (Guo et al., 2020). It is worth mentioning a dual functional lncRNA *PARTICLE* in response to low-dose irradiation. Over expressed *PARTICLE* upon irradiation recruits the PRC2 to the promoter region of *MAT2A* in a DNA-RNA triplex form, in this way to regulate *MAT2A* expression *via* methylation. The altered expression level of *MAT2A* lead to changed concentration of SAM, which further influence the methylation level of downstream genes (O'Leary et al., 2015) (Figures 3A,B). This triplex-mediated expression regulation based on interaction between lncRNA *PARTICLE* and DNA strand was further proved to be widespread in the human genome (O'Leary et al., 2017). In summary, these studies indicate that lncRNAs could regulate methylation level of downstream genes by regulating the SAM related pathway genes.

### Implications of long non-coding RNA mediated DNA methylation in drug treatment of cancer

Studies have indicated that lncRNAs could modulate gene for degradation and/or elimination of endogenous and exogenous toxins or medicines, by which they are able to exert their effects on drug metabolism and response to treatment (Table 2). For example, *LINC00261* was found to recruit DNMTs to the promoter of the dihydropyrimidine dehydrogenase (*DYPD*), which is mainly responsible for 5-fluorouracil (5-FU) degradation. Increased *LINC00261* promotes the methylation level within the *DPYD* promoter region and leads to its downregulation in esophageal cancer. As a result, 5-FU degradation is inhibited, finally results in an elevated sensitivity to 5-FU of the cancer cell (Lin et al., 2019). Similar observations were also found for the effect of *LINC01419*-*GSTP1* regulation in esophageal cancer (Chen et al., 2019). In prostate cancer, regulation of *KLF4* promoter methylation by *LINC00673* is associated with paclitaxel resistance (Jiang et al., 2020). In lung adenocarcinoma, vincristine resistance is meditated by promoter methylation of *LAMA3* induced by *LINC00628* (Xu et al., 2019). In thyroid cancer, *LINC00607* mediates doxorubicin resistance through the regulation of *CASP9* methylation (Li et al., 2021). These observations lead to the thought that the chemical drug effectiveness can be improved for better treatment by regulating the expression level of these lncRNAs.

Another possible direction for cancer treatment is to interfere with lncRNAs involved in DNA methylome regulation by using gene editing methods. One example is the lncRNA *91H* which is reasonable for inducing methylation of *CDK4* promoter, knockdown of this lncRNA could suppress the tumorigenesis of osteosarcoma (Cheng et al., 2021). Some small molecules directly interfering lncRNAs responsible for methylation regulation could also be efficient treatment targets. For instance, metformin was found to induce *H19* repression and the genome-wide DNA methylation alterations by modulating the activity of *H19—*SAHH axis, this observation provides a novel explanation for the mechanism and function of the metformin for the epigenetic regulation effect in cancer (Zhong et al., 2017). In addition, some chemical compound that interrupts the *HOTAIR—*EZH2 interaction are found to inhibit cancer cell invasion and migration, which was thought to be a potential approach for targeted therapy of cancers (Ren et al., 2019; Wang et al., 2021). In summary, lncRNAs involved in DNA methylation regulation are promising targets for applications in cancer therapy. Representative lncRNAs currently identified that are involved in DNA methylation regulation, and the associated cofactors, interaction mode, as well as target genes are listed in Table 2. This comprehensive summary revealed us a complex interaction network based on epigenetic regulatory mechanisms that remains to be further explored. In-depth analysis of non-coding RNA and other epigenetic regulatory elements including DNA methylation at the systemic level will help us to reveal the underlying mechanisms of tumor development and development, thus providing a new perspective for personalized tumor therapy.

### Role of circular RNAs in DNA methylation regulation

In recent years, circRNAs have been revealed for their crucial role during the onset and progression of human disease by their important regulatory effect. The capacity of circRNAs interact with proteins involved in epigenetic modification manifests itself the ability for the transcriptional regulation on target genes (Table 3). Examples include a circRNA termed *ACR* (autophagy related circular RNA), which directly binds to DNMT3B and block DNMT3B-mediated DNA methylation of *Pink1* promoter. *Pink1* further brings about phosphorylation of the downstream target *FAM65B*, and finally inhibits autophagy and cell death in the heart (Zhou et al., 2019). An exosome derived circRNA *circ_6790* from bone marrow mesenchymal stem cell was found to increase the nuclear translocation of CBX7, by this indirect interaction mode to recruit DNMTs and induce the methylation of *S100A11* in pancreatic ductal adenocarcinoma (Gao et al., 2022). Many circRNAs are found to regulate the expression level of DNMT genes and finally influence the downstream target methylation. For example, *hsa_circ_0012919* is downregulated in CD4^+^ T cells of systemic lupus erythematous (SLE) and results in the increased the expression of DNMT1 and finally leads to the hypermethylation of *CD70* and *CD11a* (Zhang et al., 2018). A similar example is the *circ-Amotl1*, which interacts with STAT3 and facilitate its nuclear translocation and the binding to the promoter of *DNMT3A* gene, the activated *DNMT3A* further induce miR-17 promoter methylation and decrease its expression (Yang et al., 2017). In addition, a multi-functional circRNA was found that regulate downstream methylation by different mechanisms. The circRNA derived from *FLI1* termed *FECR1* is able to recruit TET1 to the promoter of the host gene and lead to the hypomethylation *in cis*, in addition, this circRNA could also bind to the *DNMT1* promoter, where it downregulates *DNMT1* transcription *in trans*. In this manner, this circRNA regulator controls tumor growth and metastasis of breast cancer (Chen et al., 2018).

**TABLE 3:** Representative circRNAs that regulate DNA methylation of other genes in cancers and other disease.

CircRNA name	Cofactor	Interaction mode	Target	Tissue/disease	Function	References
ACR	DNMT3B	Recruit	Pink1	Myocardial ischemia/infarction	Autophagy	Zhou et al. (2019b)
Circ_6790	CBX7—DNMTs	Recruit	S100A11	Pancreatic ductal adenocarcinoma	Cell proliferation, apoptosis, metastasis, immune escape	Gao et al. (2022)
Hsa_circ_001291	DNMT1	Expression	CD11a; CD70	Systemic lupus erythematosus		Zhang et al. (2018)
Circ-Amotl1	STAT3—DNMT3A	Expression	miR-17-5p	Wound healing	Cell adhesion, migration, proliferation, wound repair	Yang et al. (2017)
FECR1	TET1; DNMT1	Recruit; expression	FLI1; SERTED2	Breast cancer	Tumor invasion, metastasis	Chen et al. (2018)
Circ_0040809	miR-515-5p—DNMT1	ceRNA		Colorectal cancer	Cell proliferation, migration, apoptosis	Mao et al. (2021)
CircSOD2	miR-502-5p—DNMT3A	ceRNA	SOCS3	Hepatocellular carcinoma	JAK2/STAT3 signaling	Zhao et al. (2020b)
CircMEMO1	miR-106b-5p—TET1	ceRNA	TCF21	Hepatocellular carcinoma	Cell proliferation, invasion, metastasis, EMT, sorafenib sensitivity	Dong et al. (2021)
CircTRIM33–12	miR-191—TET1	ceRNA	WWC3; TP53INP1; ULBP1; JHDM1D	Hepatocellular carcinoma	Cell proliferation, migration, invasion, immune evasion	Zhang et al. (2019a)
CircIBTK	miR-29b	—	Genome wide	Systemic lupus erythematosus	AKT signaling	Zhang et al. (2019a)
Circ-ATAD1	—	—	miR-34b	Acute myeloid leukemia	Cell proliferation	Wu et al. (2021b)
Circ-ATAD1	—	—	miR-10a	Endometrial cancer	Cell invasion, migration	Yang et al. (2021a)
CircFAT1	—	—	miR-21	Endometrial cancer	Cell stemness increase	Wu et al. (2021a)
CircSEPT9	—	—	miR-186	Endometrial cancer	Cell invasion, migration	Guo et al. (2022)
CircRIMS	—	—	miR-613	Esophageal squamous cell carcinoma	Cell proliferation	Wan et al. (2021)
CircSKA3	—	—	miR-1	Glioblastoma	Cell proliferation	Zhou et al. (2021a)
CircFADS2	—	—	miR-195-5p	Osteoarthritis	Apoptosis	Zhang et al. (2021b)

The ceRNA mechanism is also widely involved in the processes of methylation regulation by circRNAs. For example, *hsa_circ_0040809* regulates cell proliferation of colorectal cancer by upregulating *DNMT1 via* targeting miR-515-5p (Mao et al., 2021). Another example is from HCC, the *circSOD2* was activated by promoter modification of H3K27ac and H3K4me3, the activated *circSOD2* inhibits miR-502-5p expression and rescues miR-502-5p target gene *DNMT3A* expression (Zhao et al., 2020). Similar observations include the *circMEMO1*—miR-106b-5p—*TET1* axis (Dong et al., 2021) and *circTRIM33–12*—miR-191—*TET1* axis (Zhang et al., 2019), which play key roles for controlling cell proliferation, migration and immune evasion. This ceRNA mechanism for downstream target methylation regulation was also found during SLE development (Wang et al., 2018). Interestingly, miRNA genes are also found to be the methylation targets of circRNA regulators. For instance, the *circ-ATAD1* leads to miR-34b gene methylation in AML to increase the cell proliferation (Wu et al., 2021). This very circRNA was found to regulate miR-10a gene methylation in endometrial cancer (Yang et al., 2021). Other similar examples are also identified in many types of diseases (Table 3) (Wu et al., 2021; Zhou et al., 2021; Zhang et al., 2021; Wan et al., 2021; Guo et al., 2022). However, the detailed mechanism on how circRNA influence the methylation of miRNA gene promoters are largely unknown and remains to be further investigation.

## Concluding remarks

One of the major findings in cancer epigenetics is that genes encoding lncRNAs and circRNAs are widely connected with DNA methylome regulation in tumorigenesis. First of all, lncRNAs as well as circRNAs could be targets of DNA methylation regulation bases on the canonical epigenetic regulatory mechanism. Aberrant methylation changes at lncRNA and circRNA promoters are widely observed in a variety of physiological and pathological circumstances. Studies have identified the lncRNAs and circRNAs whose transcriptional deviation are associated with aberrant promoter methylation (Lujambio et al., 2010; Morenos et al., 2014; Boque-Sastre et al., 2015; Lu et al., 2020; Pangeni et al., 2022). On the other hand, lncRNAs and circRNAs could also regulate DNA methylation level of target genes by interaction with DNMTs or other genes involved in this process, either directly or indirectly. The study of the lncRNA-DNAm interactions has shifted our understanding of gene expression and regulation. LncRNAs usually do not function alone, but by interaction with proteins or other biomolecules to play a regulatory role in different biological processes (Teng et al., 2020; Wang et al., 2021). As a rapid way for gene expression regulation, impact on target genes by lncRNAs by re-shaping the epigenome is an effective approach to adjust cell function, through which cells can respond to diverse stimuli rapidly. Given the diversity and tissue specificity of their expression pattern, lncRNAs and circRNAs taking part in multiple cellular regulatory networks have revealed their importance in various physiological processes, and also the implications in cancer. Indeed, by using a systems biology approach, we have revealed lncRNAs that constitute master regulators of the DNA methylome in pan-cancer wide, which implicated in regulating the DNA methylation and expression levels of key genes involved in cancer development as targets (Yang et al., 2021). It is likely that lncRNAs and circRNAs establish an additional layer for transcriptional and posttranscriptional regulation defined by epigenetic landscape, which leads to reconsideration of our concept about epigenetics. As summarized in this review, evidences of the regulatory networks among lncRNAs and DNA methylation in human diseases are increasing rapidly, although many important questions regarding detailed mechanism on lncRNA regulatory complexity remain to be solved. In this context, lncRNAs could be exploited not only as specific biomarkers for early diagnosis and prognosis, but also for combined epigenetic targeting of personalized treatment of cancer.
